# Somatosensory Response to Trigeminal Stimulation: A Functional Near-Infrared Spectroscopy (fNIRS) Study

**DOI:** 10.1038/s41598-018-32147-1

**Published:** 2018-09-13

**Authors:** Christine I. Hucke, Marlene Pacharra, Jörg Reinders, Christoph van Thriel

**Affiliations:** 1 Leibniz Research Centre for Working Environment and Human Factors at the TU Dortmund, Ardeystrasse 67, 44139 Dortmund, Germany; 2 MSH Medical School Hamburg, University of Applied Sciences and Medical University, Am Kaiserkai 1, 20457 Hamburg, Germany

## Abstract

Functional near-infrared spectroscopy (fNIRS) is an optical imaging technique measuring relative hemodynamic changes in superficial cortical structures. It has successfully been applied to detect a hemodynamic response in the somatosensory cortex evoked by irritating mechanical, electrical, and heat stimulations of limbs or the face. The aim of the current study was to explore the feasibility of fNIRS to detect respective responses evoked by irritating chemical stimulations of the nasal divisions of the trigeminal nerve. In two experiments, healthy subjects were exposed to acetic acid and ethyl acetate presented using a respiration-synchronized olfactometer. Results demonstrated that fNIRS can detect a signal in both hemispheres after birhinal (experiment 1: n = 14) and monorhinal (experiment 2: n = 12) stimulations using acetic acid but not ethyl acetate. This is a first evidence that fNIRS might be a suitable imaging technique to assess chemosensory neuronal correlates in the somatosensory cortex thereby offering a new, portable method to evaluate the irritating properties of certain volatiles in an objective, nonverbal, easy, and comparably inexpensive manner.

## Introduction

Human perception of chemical stimuli, also called chemosensation, includes three sensory systems: The gustatory, olfactory, and the trigeminal system^1^, the latter being the focus of this study. The neural processes underlying chemosensation are less investigated compared to those of vision or audition. The research bias towards the ‘classical senses’ vision and audition and away from the chemosensory systems can, at least partly, be attributed to methodological challenges. Most imaging techniques require precise stimulus timing. This is straightforward if the stimulus is a picture that can be flashed on the screen in a highly controlled manner or a tone which can be switched on and off. Precise timing can become a complex issue when the stimulus is a volatile odorant. To overcome this challenge, adequate delivery devices, olfactometers, were developed which led to a considerable progress in neuroimaging research^1^. Nonetheless, central processes of chemosensation remain less explored compared to other sensory modalities. It is thus of interest to explore the capabilities and limits of measuring chemosensory-related brain activity using new imaging techniques.

Functional near-infrared spectroscopy (fNIRS) is such a possible technique. For an elaborate overview of continuous-wave fNIRS refer to Scholkmann *et al*.^2^ or Ferrari and Quaresima (2012, 2016)^3,4^. In short, similar to functional magnetic resonance imaging (fMRI), fNIRS takes advantage of the commonly known principle of neurovascular coupling. Active cells consume oxygen causing a local shortage thereof, which is coupled to an increase in blood flow and supply of oxygen-bound hemoglobin (HbO)^5^. The relative increase in HbO and decrease in deoxygenated hemoglobin (HbR) concentration can be recorded using an fNIRS system^2,6^. Light sources and detectors are placed onto the participant’s head above the target brain area. The sources continuously emit light at two discrete near-infrared wavelengths (700–900 nm) which is mainly reflected by HbR and HbO, respectively. The detectors of the fNIRS system measure the intensities of the reflected light. By transforming intensity into concentration changes using the modified Beer-Lambert law^7^ inferences about dynamic activity patterns in the underlying brain areas can be drawn. fNIRS offer a high temporal resolution that can range from milliseconds to seconds depending on the systems sampling^2,4^ and signal-to-noise ratio. fNIRS is relatively resistant to movement artifacts and enables a rather flexible and inexpensive application compared to fMRI. However, the spatial resolution is generally limited by the light penetration depth^2^ and further varies with factors such as the measurement probe arrangement, channel number and the source-detector spacing. Therefore, mostly cortical surface regions that lie close to the cranial bone can be targeted by fNIRS. Hence, hemodynamic changes in the surface areas of the occipital, temporal and frontal lobes have been explored during a wide range of visual and auditory^8^, cognitive^9,10^, and emotion^11,12^ as well as motor planning and execution^13^ tasks (often in a multimodal and multi-methodological setting^14,15^). However, is it possible to target areas related to chemosensation?

Brain regions that are related to early olfactory^16^ and gustatory processing^17^ such as the piriform cortex, the amygdala or the insula are located too far inside the brain in order to be detectable by fNIRS. Once the concentration of an airborne chemical exceeds a certain threshold (irritation threshold) the olfactory stimulus becomes an irritant since in addition to olfactory nerve fibers the trigeminal nerve is being stimulated^18,19^. Irritating or painful sensations such as ‘pungent’, ‘burning’ or ‘stinging’ are elicited by trigeminal stimulation^1,20^. Furthermore, the substance can be lateralized or localized meaning that when applied to one nostril a subject is able to determine the stimulated nostril. This is based on the assumption that humans are only able to lateralize a substance that stimulates the trigeminal nerve^21^. Thus, lateralization accuracy offers a behavioral endpoint to assess trigeminal activation. Among the cortical endpoints of the trigeminal pathway are the primary and secondary somatosensory cortices (SSC)^22–24^ which are easily accessible via fNIRS.

Even though chemosensory trigeminal related hemodynamic changes have not been studied using fNIRS, otherwise elicited irritation and pain have been investigated. Noxious mechanical, thermal, and electrical stimulations were shown to elicit hemodynamic responses in the SSC as measured using fNIRS^25–27^. In a study by Becerra and colleagues (2009)^28^ heat was applied to the participant’s forehead, more precisely to the ophthalmic division of the trigeminal nerve (V1) which is also known to be highly relevant in nasal trigeminal chemosensation. Stimulations that were perceived as ‘painfully hot’ elicited a hemodynamic response with a ‘double-peak’ in both hemispheres. The later (10–12 s) component was similarly strong in both hemispheres whereas the early component (3–7 s) and was more pronounced in the contralateral hemisphere. In an earlier study, Becerra *et al*.^25^ did find a ‘double-peak’ yet did not find any hemispheric differences. Yücel *et al*.^27^ on the other hand reported an overall stronger contralateral activation but no ‘double-peak’.

Based on these studies, it can be assumed that it is possible to use fNIRS in order to detect hemodynamic responses in the SSC that are elicited by irritating nasal trigeminal (V1 and V2) stimulation due to the inhalation of trigeminally potent chemicals^23^. It would offer the possibility of a new nonverbal, objective and relatively easy assessment of irritation. This in turn might help to evaluate chemical compounds and their risks^29^. In the current study, it was investigated whether mono- and birhinal presentation of trigeminally potent chemicals, acetic acid (AcOH) and ethyl acetate (EA), leads to hemodynamic changes in the SSC that can be recorded using fNIRS. To maximize external validity, the substances were delivered using a respiration-synchronized olfactometer^30–33^. In detail, the aim of this study was (a) to assess whether fNIRS is suitable to detect hemodynamic changes in the SSC, (b) to test the effects of birhinal and monorhinal stimulus presentation on the fNIRS signal, and (c) to compare hemodynamic changes, if found, to those reported in aforementioned published fNIRS pain studies.

## Results Experiment 1

The interval in which the maximal hemodynamic response was expected was theoretically derived from previous fNIRS and fMRI studies investigating the hemodynamic response evoked by chemical and overall irritating stimuli. Across studies the hemodynamic response to painful stimuli is reported to peak between 5 to 15 seconds after stimulus onset^25,28,34^. However, the relative contribution in pain processing of the SSC – the target area in this study – has been found to be most pronounced in the later phase of painful processing^34,35^. Therefore, the focus was set on the later phase of the reported time span and the interval of 10 to 15 seconds after stimulus onset was predefined as the temporal window of interest.

### Part A: Birhinal Stimulation

**Figure 1:**
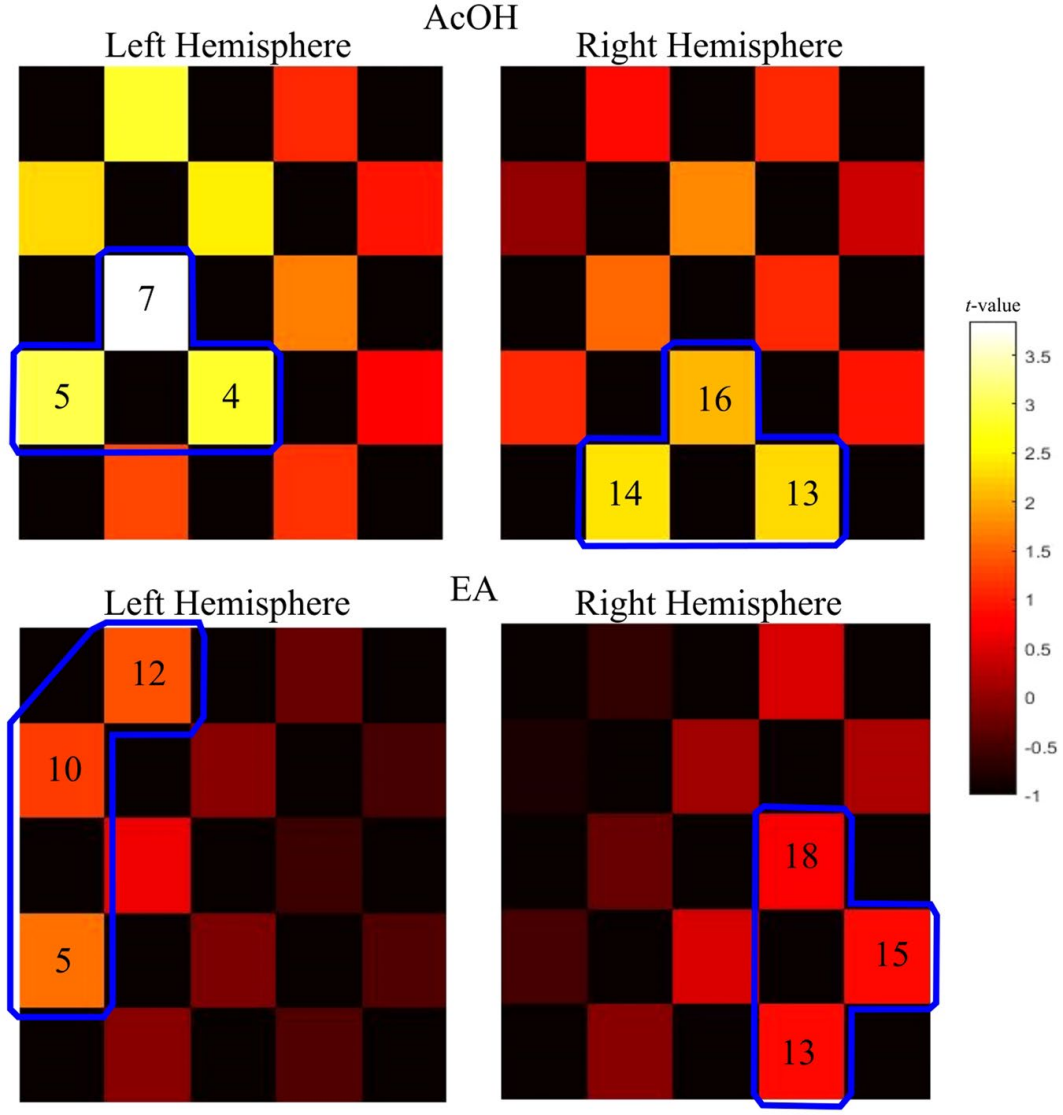
*T*-maps showing hemodynamic (HbO) changes after stimulus presentation. The color-coding represents *t*-values obtained from paired *t*-tests between interval of interest [10–15 s] and baseline. Channels selected for further analyses are marked in blue. Top figure: *t*-maps for birhinal AcOH stimulation for the left and right hemispheres. Bottom figure: *t*-maps for birhinal EA stimulation for both hemispheres, respectively.

In accordance with the method used by e.g. Yücel *et al*.^27^, HbO changes within the time interval were averaged and compared to baseline changes using paired *t-*test. Resulting *t*-values were separately visualized for AcOH and EA stimulations in form of *t-*maps (Fig. 1). These maps were used to guide the localization of activity maxima that were assumed to represent the area in the somatosensory cortex reflecting nasal trigeminal stimulation^23^.

Birhinal AcOH stimulation evoked localizable HbO maxima in both hemispheres (Fig. 1, top). In the left hemisphere, the strongest activation was found in channel 4, 5, and 7 and in the right hemisphere in channel 13, 14 and 16. Therefore, these channels were selected and averaged for further analyses of birhinal AcOH presentation. The bottom part of Fig. 1 displays *t*-maps created based on birhinal EA stimulation. Further analyses were performed on averages of channel 5, 10, and 12 in the left hemisphere and channel 13, 15, and 18 in the right hemisphere.

**Figure 2:**
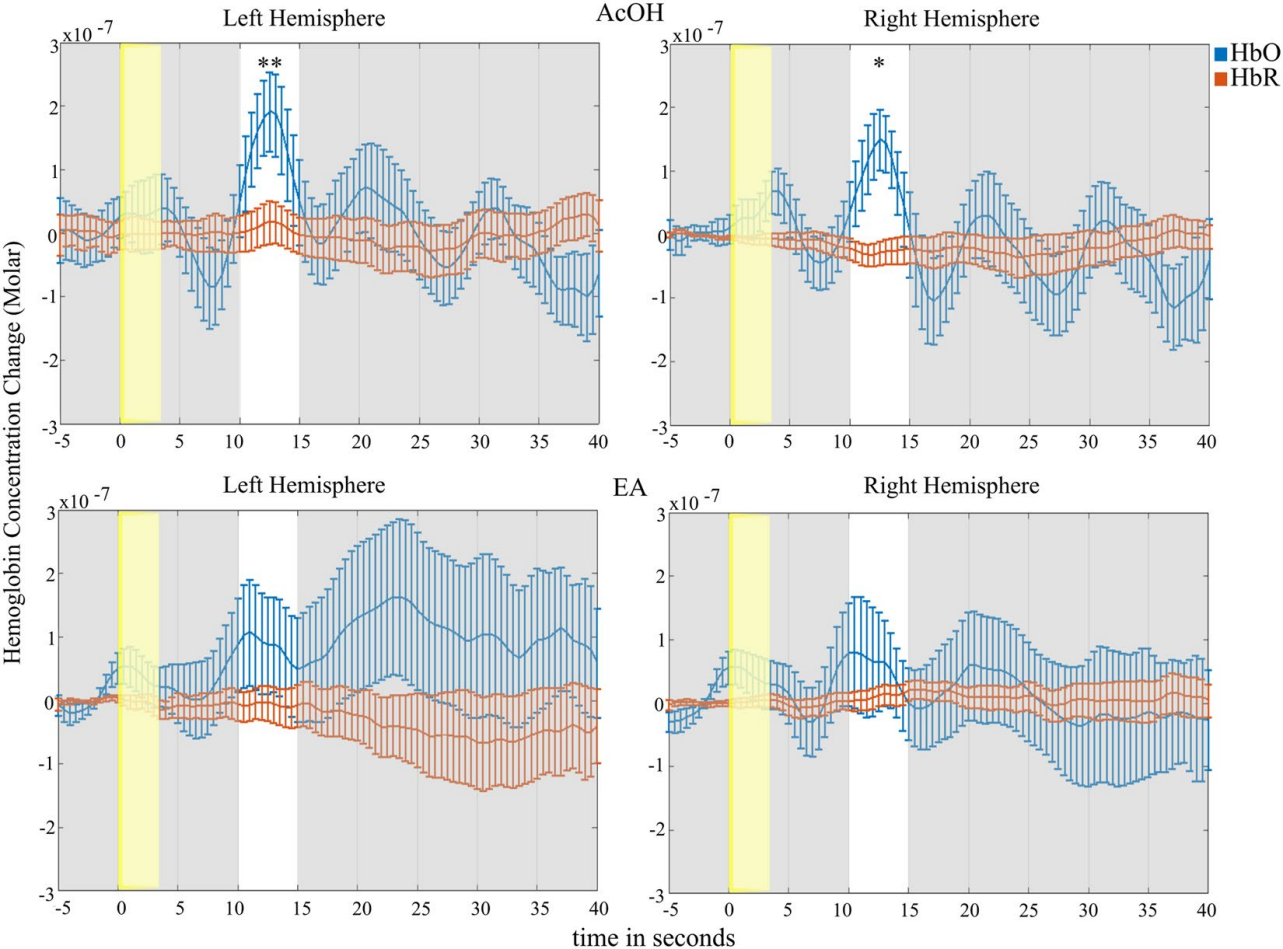
Hemodynamic response in averaged channels after birhinal AcOH (top) and EA (bottom) stimulation. Left images represent the left hemisphere and vice versa. The yellow bar indicates stimulus presentation whereas the white time window (10–15 s) marks the time interval of interest. Error bars correspond to +/− *SEM*. *p < 0.05, **p < 0.01; HbO vs baseline.

HbO concentration increase triggered by birhinal AcOH stimulation in the left hemisphere was significant (*t*(13) = 4.01, *p < *0.002) (Fig. 2, top left). The averaged HbO response in the right hemisphere was smaller, yet still significant (*t*(13) = 2.65, *p* = 0.02) (Fig. 2, top right). HbO changes due to birhinal EA stimulation in the predefined time window did not reach significance neither in the left (*p = *0.18) nor the right hemisphere (*p* = 0.41) (Fig. 2, bottom).

### Part B: Monorhinal Stimulation

#### HbO Results

The overall hemodynamic responses were considerably weaker than in Part A to a degree that none of the *t*-maps revealed strong and clustered activation peaks. Therefore, no further analyses were performed.

#### Behavioral Results

**Figure 3:**
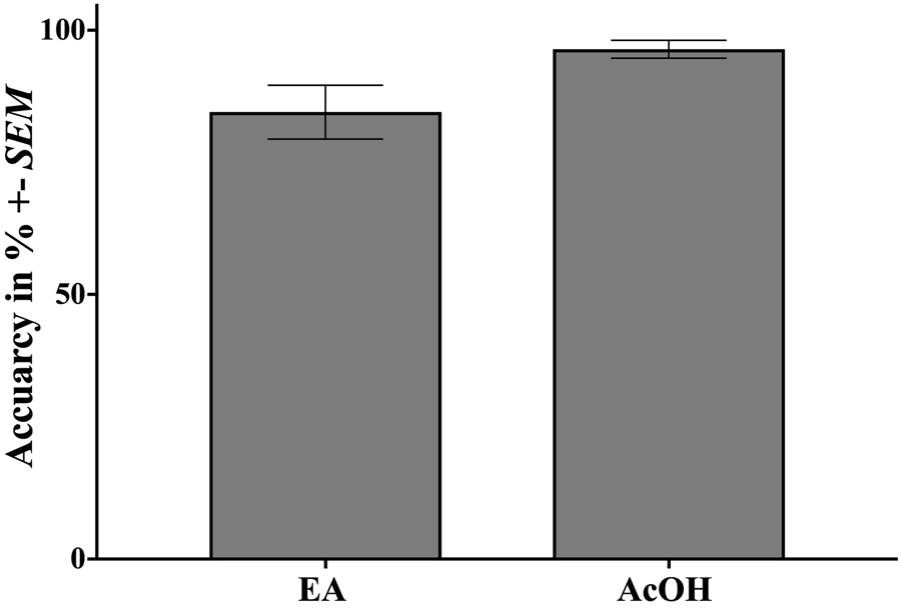
Lateralization performance for EA and AcOH expressed in accuracy (%). Error bars correspond to +/− *SEM*.

After each stimulus presentation, participants were asked to indicate the stimulated nostril resulting in a lateralization score for each substance. A paired *t*-test revealed a difference in lateralization accuracy between AcOH (*M* = 96.43%, *SEM* = 1.69%) and EA (*M* = 84.52%, *SEM* = 5.09%) with a trend towards significance (*t*(13) = 2.04, *p* = 0.06), see Fig. 3.

#### Order of Blocks

To control whether the order of blocks had an effect on the hemodynamic or behavioral results in part B, two independent sample *t*-tests were conducted including ‘order’ as an independent and ‘lateralization accuracy’ and ‘average concentration change in the selected clusters’ as dependent variables, respectively. Neither of the two *t*-tests revealed any significant effect (*p* > 0.2).

## Discussion 1

Birhinal AcOH stimulation did evoke a significant hemodynamic response in the SCC of both hemispheres that was detectable using fNIRS. This is in accordance with the expectations. Birhinal EA stimulation, however, did not evoke hemodynamic responses that significantly differed from baseline. HbO dynamics evoked by monorhinal stimulation were non-significant either.

Thus far, there is partial support for the idea that chemosensory, more precisely, trigeminally evoked hemodynamic changes in the SSC can be measured with fNIRS. However, the question arises why AcOH but not EA evoked the expected fNIRS signal when both substances, just as virtually all substances in high concentrations, are known to be trigeminally potent^36^. Furthermore, why does birhinal but not monorhinal presentation of AcOH evoke a stable significant HbO increase?

### Why Does AcOH but Not EA Stimulation Elicit an HbO Response?

The concentration of EA and AcOH were matched for intensity before the experiment using an independent subject sample. Due to the close connection of the trigeminal and olfactory systems, the trigeminal potency of two substances might differ even though the intensity is judged to be comparable. The anatomical connection of the two systems allows for a complex functional interaction including reciprocal enhancement or suppression at different levels ranging from receptor to central levels^37,38^. This in turn might result in a precept that is judged to be of certain intensity yet with unpredictable contributions of the trigeminal and olfactory systems, respectively. In a study by Scheibe, van Thriel and Hummel (2008)^39^ mucosa potentials were recorded after stimulation with AcOH, EA, and CO_2_ that were also matched for intensity. Even though the substances were not compared statistically, the results suggested that AcOH possessed a higher trigeminal potential than EA evident from higher mucosa potentials at equal levels of perceived intensity. The EA concentration in the current study exceeded the irritation threshold determined by van Thriel *et al*.^36^ as confirmed using gas chromatography (Supplement 1). However, it was lower than the threshold determined by Cain *et al*.^40^. Difference in methodology might have led to the divergent thresholds^41^. Van Thriel and colleagues (2006)^36^ determined olfactory and trigeminal thresholds using sniff bottles. Surprisingly, the same subjects detected odors and trigeminal qualities already at lower concentrations than the derived thresholds when the substances were presented with an olfactometer. However, the olfactometer used in this study might require higher EA concentrations, comparable to those determined by Cain and colleagues (2006)^40^, in order to yield a trigeminal quality that is comparable to that of AcOH. The trigeminal quality of a substance can be quantified by its lateralization accuracy. There was a trend for lateralization accuracy to be lower for EA than AcOH as well as a greater inter-individual variability of EA lateralizability (Fig. 3), further highlighting the potential lower trigeminal property of EA compared to AcOH.

A different idea of why AcOH but not EA produced a measureable fNIRS response might be that the two substances potentially interacted resulting in cross-sensitization or cross-desensitization. Jacquot, Monnin, Lucarz and Brand (2005)^42^ exposed participants to AcOH and allyl isothiocyanate (AIC) shortly after each other. Results indicated that the perception and the skin conductance response (SCR) of AIC was decreased when AcOH was presented beforehand. However, AIC did not influence the perception and SCR to AcOH. Wise, Preti, Eades & Wysocki (2011)^43^ could show that AcOH irritation threshold was increased when menthol was presented beforehand whereas the irritation threshold of AIC was decreased. These examples of complex substance interactions give rise to the possibility of AcOH altering the perception of EA due to the alternating presentation. Thus far, no study directly explored cross-sensitization or -desensitization of AcOH and EA in a comparable context. Perhaps, AcOH decreased the processing and perception of EA due to cross-desensitization. Furthermore, effects of EA on AcOH, compared to that of menthol, cannot be excluded either. Before EA and AcOH interactions are not further explored, it might be reasonable to focus on one substance at a time.

### Why Does Birhinal but Not Monorhinal AcOH Stimulation Elicit an HbO Response?

Even though the order of blocks was counterbalanced across subjects, the order might still have affected the overall results. Being stimulated with AcOH birhinally fist might have caused desensitization or habituation leading to a decrease of neural response in block B thereby decreasing the average group results. However, results of respective analyses did not reveal significant effects of presentation order on accuracy and hemodynamic responses in the monorhinal condition.

Alternatively, a lower stimulus volume in the monorhinal compared to the birhinal stimulation could have been the reason why monorhinal stimulation did not evoke a significant HbO increase due to a lower exerted trigeminal stimulation^44^. However, estimated concentration for monorhinal stimulation exceeded reported irritation thresholds^36,45^. Additionally, the lateralization accuracy of 96.43% for AcOH in this study further objects the assumption of an insufficient concentration during the monorhinal experimental part.

More likely, the non-significant results could be attributed to a low experimental power in the monorhinal part of the experiment. In the birhinal part, each stimulus condition was repeated 15 times. In the monorhinal part, however, the number of repetitions per condition had to be reduced since the fNIRS head probe becomes uncomfortable after a while and six repetitions were the best tradeoff between comfort and trial repetitions.

Furthermore, the theoretically derived (see section ‘Results Experiment 1’) rigid ad-hoc time window of interest, which seemed to be adequate for part A of the experiment, might not have been suitable for part B. The tasks in the two experimental parts differed. Whereas birhinal stimulation involves perception, the monorhinal part additionally required cognitive processes. Participants were asked to localize and remember the stimulated nostril. Therefore, the temporal windows of interest of the two experimental parts do not necessarily need to be the same. It is possible that the most relevant fNIRS signal peak evoked by monorhinal stimulation occurs even later than previously assumed from part A of the experiment and other fNIRS studies (e.g. Becerra *et al*.)^25,28^. This calls for a more flexible analysis approach in order to increase the potential gain of this explorative study.

### Shortcomings, Conclusions, and Experiment 2

Shortcomings of the first experiment become obvious. Firstly, it could only be speculated whether the non-significant activity pattern elicited by monorhinal AcOH stimulations were indeed due to low experimental power, the mismatch in analysis time interval or even a combination of factors. Furthermore, participants were not asked to rate their perceptions and thus only assumptions regarding the perceived trigeminal quality of AcOH and especially EA could be made.

The next logical step was to repeat the monorhinal part in a separate experiment adjusting the trial number. This could only be archived by dismissing one substance which additionally excluded the possibility of cross-sensitization or –desensitization. Since it could be concluded from the first experimental part that it is generally possible to evoke a hemodynamic response using AcOH, choosing AcOH over EA seemed obvious. Additionally, perceptual ratings were included in the next experiment to ensure irritation on a perceptual level. Furthermore, a more flexible, data driven approach regarding the hemodynamic peak determination was adopted (please refer to Analysis section for details). In short, instead of one time window of interest five consecutive time windows were compared to baseline. Resulting *t*-values were not only mapped but also entered a cluster analysis thereby allowing for a selection of representative channels considering the spatial and temporal dimensions of the HbO signal.

The central question was whether increasing experimental power and adapting the analysis approach could reveal a stable and possibly contralateral fNIRS signal as reported by Yücel *et al*.^27^ as an objective biomarker for lateralized stimulation and irritation.

## Results Experiment 2

### HbO Results

**Figure 4:**
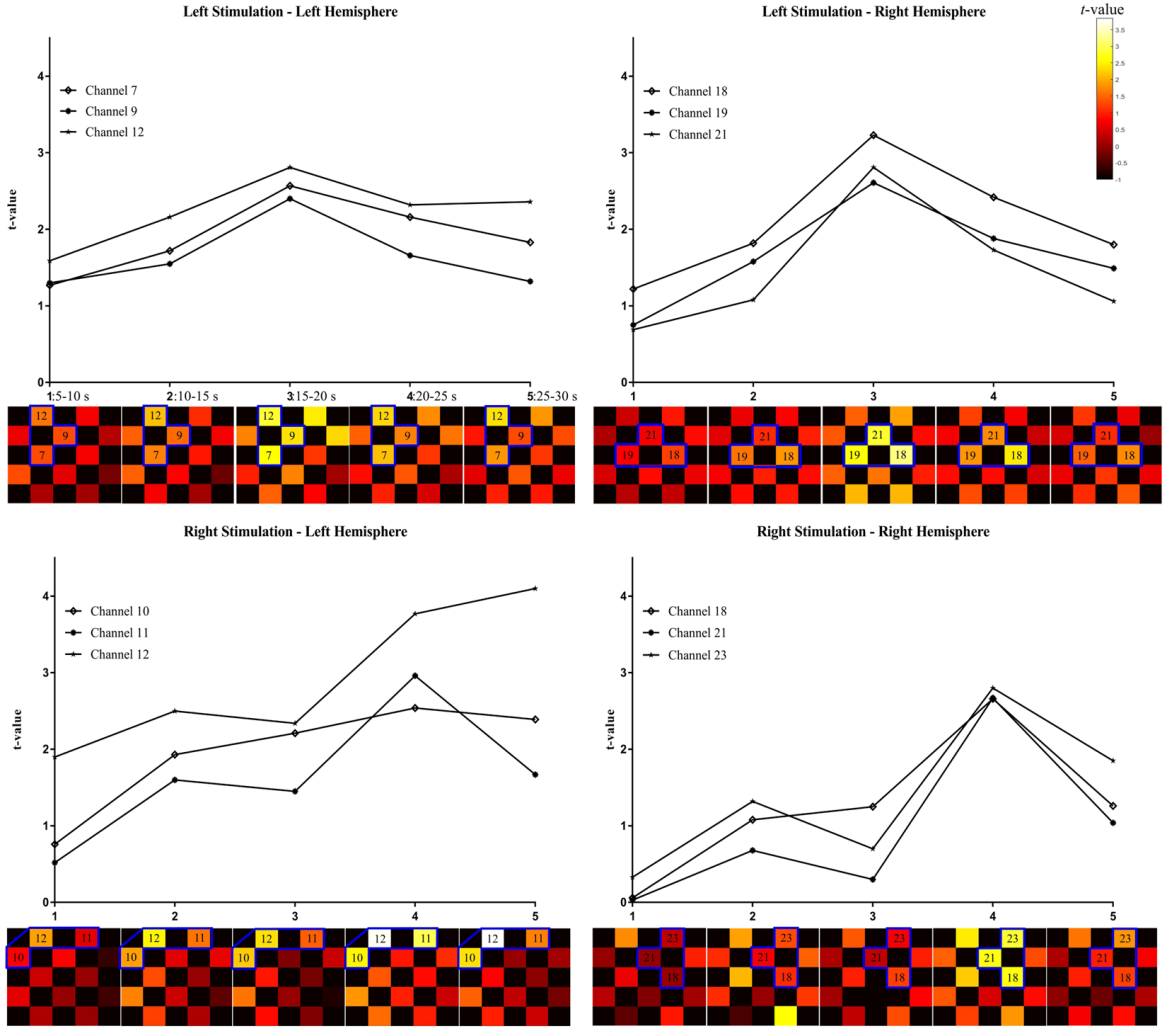
Temporal dynamics of *t*-values of channel clusters (HbO average of temporal window vs. baseline) plotted separately per condition (left-sided stimulation top figures, right-sided stimulation bottom figures) and hemisphere (left, right). The upper part of each subfigure displays the *t*-values of those channels that were most clustered, whereas the lower part displays the *t*-map of the respective time window.

After left-sided stimulation the cluster analysis revealed channel 7, 9, and 12 in the left hemisphere and channel 18, 19, and 21 in the right hemisphere to exert the strongest and most coherent signal dynamics. Right-sided stimulation triggered clustered activation patterns in the left hemisphere in channel 10, 11, and 12 and in the right hemisphere in channel 18, 21, and 22. Figure 4 displays the temporal dynamics of the channels belonging to the main clusters separately (upper half of subfigures) and within the spatial distribution of all channels in form of a *t*-map across all five temporal windows (lower half of subfigures).

**Figure 5:**
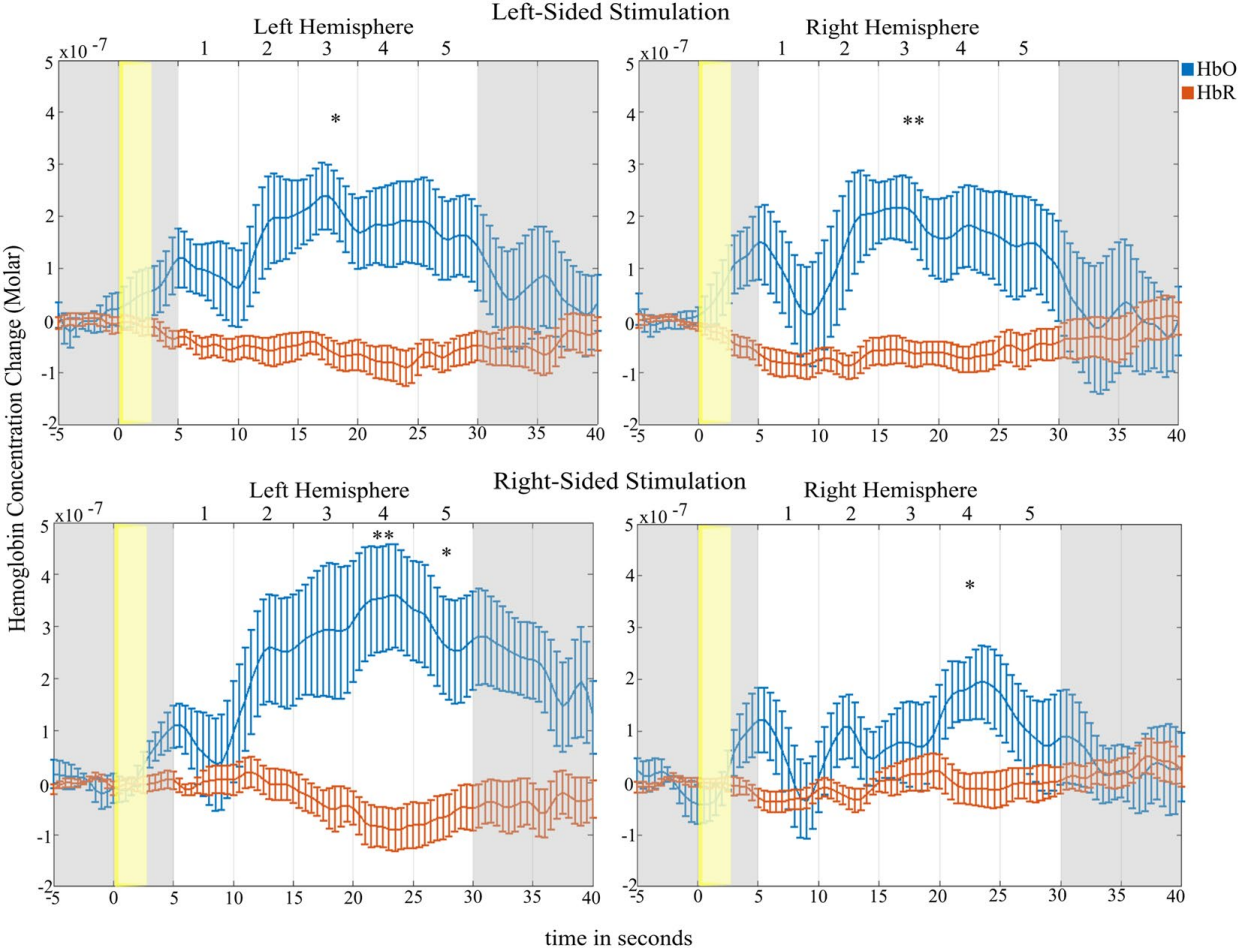
Grand average hemodynamic responses of left-sided stimulation (top) and right-sided stimulation (bottom) for the left and right hemispheres. Number 1–5 in the upper part of each subfigure denote the analyzed temporal windows. Error bars correspond to +/− *SEM.* *p = 0.01, **p < 0.01; HbO vs baseline.

The grand average HbO responses of channels forming a cluster for each condition and hemisphere can be inspected in Fig. 5. Averaged HbO values of each time window were compared to baseline using paired *t*-tests. The *p*-value threshold was Bonferroni-corrected (0.05/5 = 0.01).

Left-sided stimulation triggered HbO increase in the contralateral (right) hemisphere (*t*(11) = 3.25, *p = *0.008) 15 to 20 seconds after stimulus onset. Left-hemispheric (ipsilateral) activation was weaker, yet equaled exactly to the significance threshold (*t*(11) = 2.92, *p = *0.01). Activation in later time windows did not survive the correction for multiple comparison.

Right-sided stimulation evoked robust hemodynamic changes in the contralateral (left) hemisphere in time window 20–25 s (*t*(11) = 3.44, *p = *0.006) and 25–30 s (*t*(11) = 3.08, *p = *0.01). Activity in the ipsilateral (right) hemisphere increased to significance threshold levels (*t*(11) = 3.09, *p* = 0.01) in the fourth time window (20–25 s).

Comparing contra- to ipsilateral activation revealed no hemispheric difference after AcOH stimulation of the left nostril in neither time window (*p* > 0.5). After right-sided stimulation a difference was found that, however, did not survive the *p*-value correction (time windows 1, 3, and 5: *t*(11) = 2.24, 2.22, 2.25, *p* = 0.047, 0.049, 0.046, respectively; time windows 2 and 4: 0.07 < *p* < 0.1).

### Behavioral Results

The mean lateralization accuracy (*M* = 87.83%, *SEM* = 2.83%) was significantly higher than chance level (*t*(11) = 4.54, *p* = 0.001) throughout both blocks (block difference: *t*(11) = −0.33, *p* = 0.75). Comparing experiment 1 and 2 participants performed significantly better in the first experiment (*t*(24) = 2.7, *p* = 0.013).

**Figure 6:**
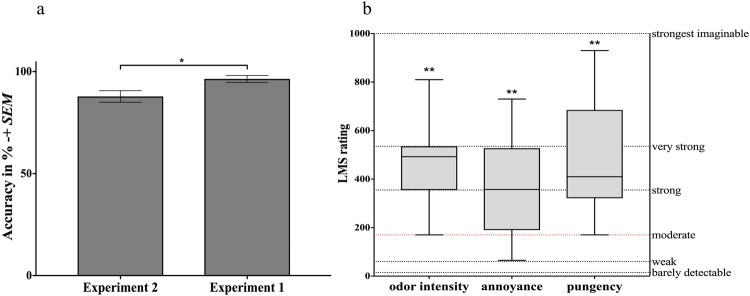
Behavioral data including (**a**) lateralization performance expressed in accuracy (%)+/− *SEM* for experiment 1 and AcOH of experiment 2 and (**b**) LMS ratings for ‘odor intensity’, ‘annoyance’, and ‘pungency’. The dotted red line indicates the minimum rating required to be categorized as ‘irritating’. *p < 0.05, **p < 0.01.

Labeled Magnitude Scale (LMS) scores were significantly higher than ‘moderate’ (odor intensity: *t*(11) = 5.83, *p < *0.001; annoyance: *t*(11) = 3.66, *p = *0.004; pungency: *t*(11) = 4.75, *p = *0.001) confirming expected perceptions were triggered by AcOH stimulations. Lateralization accuracy and LMS ratings can be inspected in Fig. 6.

## Discussion 2

The current study demonstrates that it is possible to use fNIRS to detect a hemodynamic response over the SSC evoked by birhinal (experiment 1) as well as monorhinal (experiment 2) AcOH stimulation. *T*-maps and cluster analyses enabled the selection of most representative channels to observe the temporal development of the hemodynamic changes. Averaging these channels rendered a statistically significant response with different temporal dynamics for bi- and monorhinal stimulations. Furthermore, monorhinal stimulation did not evoke a significantly stronger response in the contralateral compared to the ipsilateral hemisphere. Lastly, the lateralization accuracies for AcOH differed between the two experiments. These aspects will be discussed in the following sections.

### Temporal Differences: Bi- vs. Monorhinal Stimulation

The temporal dynamic of the fNIRS signal differed after birhinal and monorhinal stimulation. Birhinal stimulation evoked a significant response lasting around 5 seconds peaking at 7.5 seconds post-stimulus. Monorhinal stimulation, on the other hand, evoked an HbO increase lasting for up to 30 seconds peaking between 15 and 25 seconds. A similar phenomenon has been observed in an fMRI study^46^ in which participants either compared or solely perceived consecutive nasal trigeminal stimulations. The ‘perceive’ condition was similar to the birhinal stimulation in experiment 1 resulting in less pronounced activations. The ‘compare’ condition consisted of similar task dimensions as the current monorhinal condition such as attention, decision-making, and working memory, evoking a greater activation pattern. Further studies are needed to experimentally disentangle the differences occurring due to bi- versus monorhinal stimulation as well as the respective sensory, cognitive, and memory components of the chemosensory-related fNIRS signal.

### Contralaterality of the fNIRS Signal

Monorhinal stimulation triggered a significant hemodynamic increase in the contralateral hemispheres. A slightly weaker response was detected in the ipsilateral hemispheres. However, left-sided stimulation did not evoke significantly stronger activity in the contralateral (right) compared to the ipsilateral (left) hemisphere. A trend towards a stronger activity in the contralateral (left) hemisphere was found after right-sided stimulation. Thus, our results are more in line with Becerra^25^ who did not find hemispheric difference either, or only in the early phase of the signal^28^, and in contrast with Yücel *et al*.^27^ reporting a greater response in the contralateral hemisphere. The question arises why some studies find whereas others do not report a stronger contralateral signal after irritating stimulations.

The use of different fNIRS systems may partly explain these ambiguous findings. In general, fNIRS is susceptible to systemic physiological processes such as blood flow changes in the scalp (for a neat overview refer to^2^ section ‘Classification of signal components’). Newer fNIRS systems include short separation channels that are specifically susceptible to hemodynamic changes in the scalp, making it possible to regress out these effects. Studies that did not find hemispheric differences used fNIRS systems without short separation channels^25,28^. Yücel and colleagues^27^, who used a system including said channels, did find a stronger activity in the contralateral hemisphere. Thus, short separation channels may markedly increase the sensitivity of fNIRS to reveal subtle hemispheric differences. In a separate publication, Yücel *et al*.^47^ directly demonstrated the effect of a short separation regression. Analyses with and without short separation regression yielded significant hemodynamic responses, however, differing in their morphologies. Performing no short separation regression yielded a hemodynamic response whose morphology closely resembled that of the current and other studies not using short separation channels either^25,28^. This resemblance, on the one hand, suggests that a certain portion of recorded activity, indeed, originated in a localized and functionally involved brain region. At the same time, it also suggests that the signal from the SSC may have been affected by extracortical signals to some degree hampering the detection of a slightly stronger contralateral signal. The potential benefit of a short separation regression becomes evident when the role of the trigeminal system is considered. Trigeminal processes guide the detection of and protection from inhaling potentially harmful volatiles^19,38,48^, which has been reported to evoke systemic reactions^49^.

A trend towards contralaterality was found after right-nostril stimulation. No such pattern was observed when the left nostril was stimulated. A reason for the missing trend may be an overall stronger involvement of the left hemisphere, independent of stimulated side. Indeed, after birhinal stimulation a slightly stronger response was detected in the left hemisphere. Boyle *et al*.^50^ contrasted left versus right-nostril stimulation with CO_2_ in an fMRI setting, revealing more responsive brain areas in the left hemisphere. However, the reversed pattern or a classical contralaterality was also reported after birhinal and monorhinal application of CO_2_^51,52^ and during a more general processing of painful stimulations^51–57^. Psychophysical chemosensory studies found a right-nostril dominance with regard to lateralization speed^58^ and accuracy^59^ perhaps being related to the here found left-hemispheric dominance. Future studies might investigate this relationship and thereby putting the contradicting results into a clearer perspective.

### Lateralization Accuracy Differences

Participants lateralized the monorhinal AcOH presentation with a significantly higher accuracy in the first experiment compared to experiment 2. The better lateralization performance in experiment 1 might be due to the aforementioned substance interaction where the alternating stimulation with EA may have enhanced the perception and thus the localizability of AcOH. Alternatively, it is possible that participants habituated to the stimulation of AcOH in experiment 2 due to repeated presentation of the same substance^60^. The decreased perception could have complicated the localization. Habituation may have been prevented in experiment 1 due to an dishabituating effect by the alternating presentation of two substances as has been observed for olfaction^61^. Lastly, participants in experiment 1 may have first identified the substance before deciding on the side of stimulation, even though the identity of substances were not part of the questions. The time needed for the identification might have served as a temporal buffer delaying the localization decision until the slowly increasing trigeminal percept was fully developed. Since only AcOH was used in experiment 2 the identification process was omitted inevitably, potentially leading to more errors. These explanations are not mutually exclusive.

### Final Conclusion

This is the first study demonstrating the feasibility of fNIRS in the context of trigeminal chemosensory perception. fNIRS can be used to detect hemodynamic responses in the SSC after the birhinal as well as monorhinal presentation of AcOH. Thus, fNIRS might offer a portable method to evaluate the irritating properties of certain volatiles in an objective, nonverbal, easy, and comparably inexpensive manner.

## Methods

### Participants

Exclusion criteria for the two independent experiments and the pilot study included pregnancy, history of asthma, upper airway diseases (e.g. chronic rhinosinusitis), migraine or psychiatric and neurological or diseases, and reports of acute illness. Before participation, the participants passed (a) a lung function test (FEV 1 > 80%, MasterScope TP, JAEGER/CareFusion, Höchberg, Germany), (b) a test of normal olfactory function^62^ (Sniffin’ Sticks, Burghart Messtechnik GmbH, Wedel, Germany) and (c) a nasal flow rate exam using active anterior rhinomanometry (RHINO-SYS, Happersberger otopront GmbH, Hohenstein, Germany). Table 1 displays descriptive data of the participants of both experiments included in the data analysis.

**Table 1:** Demographic data of subjects who were included in the final analysis of experiment 1 and 2.

	Experiment 1	Experiment 2
N (f/m)	14 (7/7)	12 (7/5)
Age *M (SD)*	26.43 (3.52)	25.08 (4.30)
Sniffin’ Sticks ID^a^ *M (SD)*	13.57 (1.00)	13.08 (1.16)
FEV 1 *M (SD)* in %	104.24 (11.76)	95.76 (10.66)

*Note*. The samples did not differ statistically in any of the listed criteria (*p* > 0.05).

^a^ID: Identification subtest

#### Experiment 1

Initially, 21 right-handed participants were recruited to take part in experiment 1 of whom seven participants were excluded from data analysis. Of those seven, six participants were excluded due to a poor lateralization accuracy (< chance cut off) during part B of the experiment for the used chemicals. A poor lateralization accuracy indicates that the stimulation did not activate trigeminal nerve fibers^21^ and thus the somatosensory cortex. Inspections of the anterior rhinomanometry measurements revealed decreased nasal flow rates and thus nasal obstruction throughout the experiment for these six participants. One further participant was excluded since none of the control ‘air’ trials were correctly identified but instead erroneously categorized as chemical stimuli. Refer to the left column in Table 1 to inspect the descriptive data of the remaining 14 subjects that were included in the final analysis.

#### Experiment 2

An independent sample of 14 participants were recruited of which 12 participants entered the final analysis (Table 1, right column). One participant was excluded due to a poor lateralization accuracy (< chance cut off) that was traced back to a decreased nasal air flow. The second participant was excluded due to low perceptual ratings (pungency rating < ‘moderate’).

### Materials

#### Stimuli

Experiment 1: Acetic acid (AcOH; CAS: 64-19-7) and ethyl acetate (EA; CAS: 141-78-6) were chosen as trigeminal stimuli due to their well described chemosensory effects^36^. The liquid concentrations were 30% v/v for AcOH and 7.4% v/v for EA. Distilled water was used as solvent for both substances and for the control condition ‘air’. Gas chromatography was used to verify that the stimulus concentrations in the gas phase as presented by the olfactometer exceeded previously reported trigeminal thresholds^36^ throughout the experiment. For details of the gas chromatography analysis, see Supplement 1.

Experiment 2: AcOH in the same concentration as in experiment 1.

Pilot Study: In order to match the substances for intensity 10 participants (Age: *M* = 25.8, f/m = 7/3) who passed the participation requirements were presented with EA and AcOH to the left or right nostril using the same olfactometer as in the main part of the study (see below). The order of substances and nostril side were semi-randomized summing to twenty presentations, five presentation of each substance per nostril, respectively. Inter-trial-interval (ITI) was ~45 seconds. After each presentation, the subject indicated the stimulated nostrils verbally. Based on these answers a lateralization score (correctly lateralized in %) was calculated. At the end of the experiment, participants were asked to evaluate the intensity of the two substances in their own words and to indicate if one substance was perceived as more intense in terms of olfactory and trigeminal perceptions. Predetermined criteria for the chosen concentration were (a) equal perceptual intensities and (b) lateralization accuracy above chance level (EA: *t*(9) = 5.3, *p* < 0.001; AcOH: No statistics possible since accuracy was 100% in all subjects).

#### Olfactometer

For the pilot study and both experiments the same computer-controlled (PsychoPy^63,64^), commercially available olfactometer was used to deliver the stimuli. It allows for respiration-synchronized bi- and monorhinal stimulation (NeuroDevice, Version 2, Warsaw, Poland). The device is composed of two units: The control unit and the repository unit. The control unit is located in the control room and the repository unit located in the experimental room. The control unit contains a pump which supplies the repository unit with clean air (2.5 l/min) via a pipe containing ten independent airways that passes through a waveguide into the experimental room. The repository unit consists of two separate yet identical components thereby enabling independent stimulation of the left and right nostril. Half of the airways are part of the left and the other half of the right repository component. For each component, one of the five airways (default line) ensures constant clean airflow into the participant’s nose throughout the experiment and therefore bypasses the rest of the device. The remaining four airways reach into tightly sealed 12.5 ml screw-topped test tubes containing 2.5 ml of the stimulus solutions or distilled water. A hose leads out of each test tube to the component’s outlet. The outlets of the left and right compartment are connected to the left and right hose of a disposable nasal cannula, respectively. A clip disconnects both sides of the nosepiece of the cannula and serves as an additional stabilizer of the cannula prongs inside the participant’s nostrils without altering the natural nasal breathing.

The olfactometer is connected to a respiration belt and to the fNIRS recording PC. Whenever a stimulation is due over the course of the experiment the point of maximal exhalation and inhalation onset is registered and the olfactometer triggers the stimulation which is logged in the continuous fNIRS recording. The device switches the airflow from the default line to the airway connected to the test tube containing the stimulus substance. The gaseous content of the headspace is being pushed out of the test tube into the nasal cannula. After the stimulus time has lapsed, the device switches the airflow back to the default line. This switching technique integrates the stimulus into the constant air stream and thereby prevents a drop of air pressure and a consequent mechanical trigeminal stimulation inside the participant’s nose. Immediately following each stimulus, a second switch from the default line to a separate test tube containing distilled water is initiated in order to ensure that the device and hoses are clean from any stimulus residue.

The repository setup was customized to ensure constant and high concentration in the headspace of the test tubes. The components containing the solutions were mounted on a magnetic stirrer (IKAMAG REO, IKA®-Werke GmbH & CO. KG, Staufen, Germany) and a Teflon®-coated magnetic stir bar was inserted into each test tube. Set to 600 rpm a constant movement of the solutions enforced a continuous release of molecules into the headspaces throughout the experiment.

#### fNIRS System

**Figure 7:**
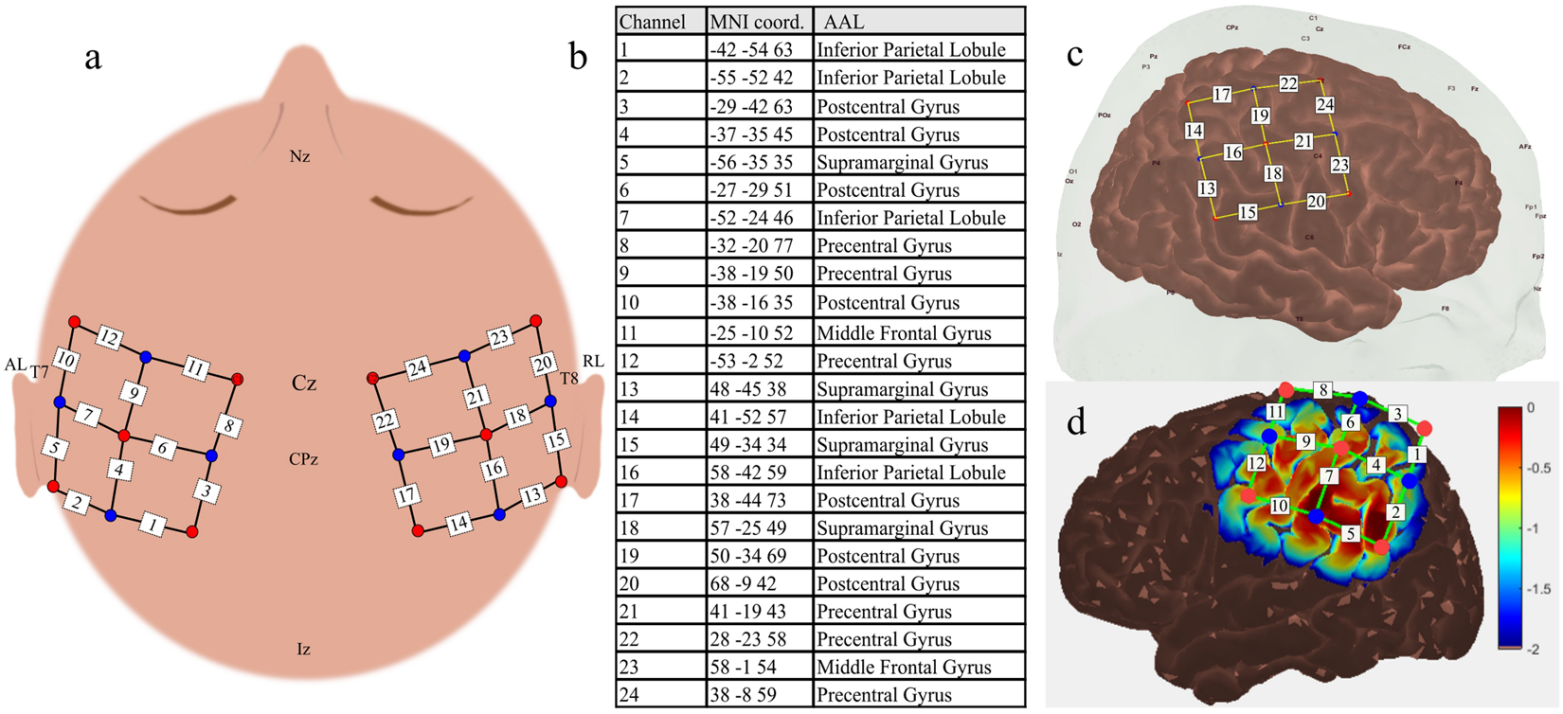
fNIRS measurement setup. (**a**) Probe arrangement and corresponding channels (1–24) as referenced to international 10/10 positions. Red: Sources; Blue: Detectors; White numbered boxes: Channels. (**b**) Channel projections onto underlying structures performed in AtlasViewer resulting in coordinates in standard MNI space and corresponding brain labels (Automated Anatomical Labeling, AAL). (**c)** Probe position planning in AtlasViwer software. (**d**) Sensitivity profile of the constructed probe setup as simulated in AtlasViewer using the built-in tMCimg software. Sensitivity is displayed on a logarithmic scale spanning two orders of magnitude (0 to −2) in arbitrary units with values around ‘0’ represent high sensitivity of the channels to the underlying neural structures and more negative values represent a relatively decreasing sensitivity.

A multichannel continuous wave fNIRS system (Hitachi-ETG 4000, Hitachi Medical Corporation, Tokyo, Japan) was used to record hemodynamic changes at a sampling rate of 10 Hz. The measurement setup comprised 10 sources emitting light at wavelengths 695 and 830 nm and eight detectors that were alternatingly mounted on a 2 × 3 × 3 head probe. All sources were paired with up to four adjacent detectors within a distance of 30 mm forming a channel. In total, the measurement list consisted of 24 channels (Fig. 7a).

Probe position planning and channel projection onto underlying cortical structures (Fig. 7b,c) were performed using the AtlasViewer^65^ in order to ensure appropriate placement over the SSC. Using a Monte-Carlo photon transport software^66^ included in AtlasViewer the probe sensitivity to the target brain structure was further validated (Fig. 7d). The positioning was guided by the international 10/10 positions: Nz, Cz, CPz, Iz, C1, C2, T7, T8, AL, and AR. The setup was kept the same for both experiments.

### Protocol

The study protocols were approved by the Ethics Committee of the Leibniz Research Centre for Working Environment and Human Factors at the TU Dortmund and were carried out in accordance with the relevant guidelines and regulations. Participants were fully briefed about possible effects of the substances (e.g. malodor and irritation) before the experiment started. Informed consent was obtained from all participants.

To minimize light interference with the fNIRS signal, both experiments were conducted in the same darkened room. The subject was seated in front of a computer monitor in a comfortable distance and position. After the fNIRS head probe and olfactometer were fixated, the participant was instructed to breathe as naturally and constantly as possible, especially when a stimulation was perceived and to move as little as possible throughout the experiment.

#### Experiment 1

The experiment was separated into two parts, whose order was counterbalanced across participants. The stimuli were presented birhinally in Part A and monorhinally in Part B.

In Part A, 45 stimuli were grouped into 15 blocks. Each block contained one trial of each birhinal stimulation condition. The stimulus order within a block was randomized. Each trial started with a waiting period until an inhalation was detected by the system which triggered an inspiration-locked 3 second stimulation. The trails were separated by ITIs of ~45 seconds. An ITI jitter was ensured by the individual stimulation onset locked to an inhalation.

In Part B, 30 stimuli were grouped into six blocks containing five trials each. Again, each trial started with a waiting period until an inhalation was registered. This time, however, a monorhinal 3 second stimulation followed by a post-stimulus waiting period of 30–45 seconds was triggered. Additionally, participants were instructed to identify and remember the stimulated side until the question ‘Did you perceive a stimulus? If yes, please indicate the stimulation side. LEFT, NO, RIGHT’ appeared on the screen. After responding by means of a mouse click, the consecutive trial was initiated after 40 seconds.

The two experimental parts were separated by a break during which the lights were turned back on and a second anterior rhinomanometry was conducted to check whether the nasal airflow indicated possible nasal obstructions. The minimum length of the break was set to 10 minutes, however, the participants were left to decide when they felt comfortable to start the consecutive block.

After both experimental parts and before the participants were thanked and seen off, a final anterior rhinomanometry was conducted. Furthermore, they received either 30 € for the total participation time of 3 hours or participation credits as part of university class.

#### Experiment 2

Again, the experiment was split into two parts, however, this time both blocks were identical. Each part contained three blocks à six randomized stimulations of the three conditions (AcOH left, right, air). Thus, both parts combined each stimulus was repeated 12 times. Trial procedure, break, and overall participation time as well as remuneration was kept the same. During the break participants were asked to rate their perception (intensity, annoyance, and pungency) on a logarithmically scaled Labeled Magnitude Scale ranging from ‘barely detectable’ to ‘strongest imaginable’ (numeric scale: 1–1000)^67^.

## Analysis

### fNIRS Analysis

#### Preprocessing

The same preprocessing stream was used for experiment 1 and 2. Data preprocessing as well as hemodynamic response function (HRF) estimation was performed using the Windows version of Homer2^68^ run in Matlab (R2016b, MathWorks, Inc., Natick, US) and followed a data processing stream used in a recent pain study^69^. In order to convert the raw data into a Homer2-compatible format an adapted version of a freely available Matlab script (by Dr. Dewey https://www.nitrc.org/projects/hitachi2nirs) was used. The signal was converted into optical density changes on which motion artifact detection and stimulus rejection (if appropriate) as well as low pass filtering at 0.5 Hz was performed. Next, optical density was transformed into HbO and HbR concentration changes using the modified Beer-Lambert law^7^ with 6 as partial path length factor for both wavelengths. Ordinary least squares method was applied to solve the general linear model using consecutive Gaussian functions with a width of 1 standard deviation and a temporal mean separation of 1 second over a time span of 45 seconds [−5–40 s] with respect to the stimulus onsets. A 3^rd^ order polynomial drift correction was chosen.

#### HbO Analysis

Statistical analyses were performed on HbO concentration changes using custom written Matlab scripts. In order to control for sinusoidal patterns in the HbO signal caused by the respiration locked stimulation, the condition ‘air’ was subtracted from each conditions timeline to perform a baseline correction. This baseline correction further controlled for activation that might have been evoked by the pure act of breathing^70^ even though such activations are more frequently investigated and found in olfactory regions^32,71,72^ or the cerebellum^73^. Thus, only hemodynamic changes exceeding the naturally occurring fluctuations due to respiration were considered in the analysis. Significance level was set to 0.05 and Bonferroni-corrected where appropriate.

Experiment 1: Experimental Part A (birhinal stimulation) and B (monorhinal stimulation) were analyzed separately yet using the same approach. Firstly, paired *t*-tests comparing the HbO response of each channel during time interval of interest [10–15 s] to the pre-stimulus ‘baseline’ interval [−5–0 s] were used to create *t*-maps to localize the activity maximum in the left and right hemisphere, respectively. Secondly, three channels in each hemisphere were selected based on highest activity for further analyses. Note, the selection was based on the highest *t*-values independent of significance level. Hence, analyses were performed even if the chosen channel did not quite reach the significance threshold (*p* < 0.05) but a relatively higher activity level than baseline. Thirdly, the HbO changes of the selected channels were averaged resulting in a grant HbO response which was analyzed by conducting paired *t*-tests between baseline and the time interval of interest.

Experiment 2: Hemodynamic changes for each channel were first split into consecutive 5-second intervals; baseline: −5–0 s, temporal windows 1: 5–10 s, 2: 10–15 s, 3: 15–20 s, 4: 20–25 s, and 5: 25–30 s post-stimulus. Secondly, paired *t*-tests between the five time windows and baseline averages were conducted, for all channels and for the two conditions separately. Thirdly, channels attributed to one hemisphere and the respective five *t*-values were entered in a cluster analysis based on Euclidean distance (SPSS 25, Armonk, NY: IBM Corp.), again for both conditions separately. Next, the HbO response of one distinct cluster of neighboring channels per condition and hemisphere was averaged and analyzed using paired *t*-tests (baseline against each time interval).

### Behavioral Analysis

#### Experiment 1

A paired *t*-test was used to compare lateralization accuracies of AcOH and EA.

#### Experiment 2

A one-sample *t*-test as used to test if the lateralization accuracy surpassed chance level. Additionally, the lateralization accuracies of the two blocks were compared using a paired *t*-test. Furthermore, an independent sample *t*-test was used to compare the lateralization accuracies for AcOH across experiments.

## Electronic supplementary material

Supplement 1

## Data Availability

The datasets generated and/or analyzed during the current study can be made available from the corresponding author on reasonable request.
