# Supplementary material for: Somatosensory Response to Trigeminal Stimulation: A Functional Near-Infrared Spectroscopy (fNIRS) Study
Source: Sci Rep. 2018 Sep 13;8:13771. doi: 10.1038/s41598-018-32147-1 (PMC6137212; doi:10.1038/s41598-018-32147-1)
Supplement: Supplementary file 1 — Supplement 1 [file 41598_2018_32147_MOESM1_ESM.pdf]

Somatosensory Response to Trigeminal Stimulation: A Functional Near-Infrared Spectroscopy (fNIRS) Study

Christine I. Hucke*, Marlene Pacharra, Jörg Reinders, and Christoph van Thriel

* Corresponding Author

Supplement 1

Gas chromatography analysis (GCA)

The gas phase concentrations of EA and AcOH were determined by means of gas chromatography (GC) using flame ionization detection (GC-FID). For the analyses, the gas chromatograph (SHIMADZU, GC14A, column (CP PoraBOND: 10m*0.32mm*5µm) was calibrated with defined amounts of ethyl acetate and acetic acid. Calibration curves were obtained and accepted when the coefficient of determination (R^2) was at least 0.98.

Ethyl Acetate. Three independent measurements, each simulating one experimental run, were conducted. A run consisted of 15 stimulus presentations every 90 seconds. The 1st, 5th, 9th, 12th, and 15th stimulus was collected into a separate Tedlar® gas sampling bag that was connected to the nosepiece of the nasal cannula. Two samples were taken from each Tedlar® bag and injected into the gas chromatograph for the analysis of 250µl each. The variation coefficient across all measurements was ~10%.

Acetic Acid. Due to the lower vapor pressure of AcOH Tedlar® gas sampling bag were not suitable for the compound. Therefore, AcOH was sampled directly from the headspace of a test tube. The outlet of a test tube was attached to the CG and nine samples were drawn out of the test tube every 10 minutes. The GC parameters were kept the same. The variation coefficient was ~5%.

Average concentration inhaled by a subject

An average person inhales about 500 ml of air in a single breath during normal breathing. Given the olfactometer flow of 2.5 l/m (1.25 l/m per side) and the stimulus duration of 3 sec, a stimulus volume of 125 ml was estimated for birhinal stimulation and 62.5 ml for monorhinal stimulation. Thus, the concentrations as assessed by the GCA needed to be adjusted accordingly; $\frac{1}{4}$ for birhinal stimulation and $\frac{1}{8}$ for monorhinal stimulation. The concentrations as measured by the GC and the derived stimuli concentrations reported in parts per million (ppm) can be inspected in Table S1.

Table S1

Concentration as resulting from the GC measurement and calculated concentration as inhaled by a subject when being birhinally and monorhinally presented with acetic acid or ethyl acetate, respectively.

	Acetic Acid	Ethyl Acetate
GCA (*M*)	462 ppm	12,800 ppm
Birhinal Stimulation[a]	115.5 ppm	3,200 ppm
Monorhinal Stimulation[b]	57.75 ppm	1,600 ppm

[a] GCA/4

[b] GCA/8
